# Framing and Tailoring Prefactual Messages to Reduce Red Meat Consumption: Predicting Effects Through a Psychology-Based Graphical Causal Model

**DOI:** 10.3389/fpsyg.2022.825602

**Published:** 2022-02-09

**Authors:** Patrizia Catellani, Valentina Carfora, Marco Piastra

**Affiliations:** ^1^Department of Psychology, Catholic University of the Sacred Heart, Milan, Italy; ^2^Computer Vision and Multimedia Lab, University of Pavia, Pavia, Italy

**Keywords:** message framing, prefactual messages, meat consumption, regulatory focus, deep reinforcement learning, dynamic bayesian network, soft clustering, message tailoring

## Abstract

Effective recommendations on healthy food choice need to be personalized and sent out on a large scale. In this paper, we present a model of automatic message selection tailored on the characteristics of the recipient and focused on the reduction of red meat consumption. This model is obtained through the collaboration between social psychologists and artificial intelligence experts. Starting from selected psychosocial models on food choices and the framing effects of recommendation messages, we involved a sample of Italian participants in an experiment in which they: (a) filled out a first questionnaire, which was aimed at detecting the psychosocial antecedents of the intention to eat red/processed meat; (b) read messages differing as to the framing of the hypothetical consequences of reducing (gain, non-loss) versus not reducing (non-gain, loss) red/processed meat consumption; (c) filled out a second questionnaire, which was aimed at detecting participants’ reaction to the messages, as well as any changes in their intention to consume red/processed meat. Data collected were then employed to learn both the structure and the parameters of a Graphical Causal Model (GCM) based on a Dynamic Bayesian Network (DBN), aimed to predicting the potential effects of message delivery from the observation of the psychosocial antecedents. Such probabilistic predictor is intended as the basis for developing automated interactions strategies using Deep Reinforcement Learning (DRL) techniques. Discussion focuses on how to develop automatic interaction strategies able to foster mindful eating, thanks to (a) considering the psychosocial characteristics of the people involved; (b) sending messages tailored on these characteristics; (c) adapting interaction strategies according to people’s reactions.

## Introduction

Red/Processed Meat Consumption (from now on RPMC) has substantial effects on people’s health, such as an increased likelihood of developing various cancer and type 2 diabetes (Misra et al., 2018; Bianchi et al., 2019). For this reason, health authorities (e.g., World Health Organization, 2015) have recommended eating no more than 3 ounces (85 g) per meal, no more than a couple of times a week (e.g., Bach-Faig et al., 2011). However, according to a report by World Population Review (2021), the average meat consumption in the world is still too high. Despite multiple government and social initiatives aimed at promoting meat reduction, people still face many difficulties in following recommendations in this direction (Stoll-Kleemann and Schmidt, 2017).

Is it possible to think of a communication on healthy eating that is effective, personalized and at the same time easily addressed to many people? Collaboration between social psychology and Artificial Intelligence (AI) can help achieve this goal. On the one hand, social psychology has developed consolidated models of the psychological factors that underlie food choices. On the other hand, AI can, starting from psychosocial models, assess their predictive capacity, as well as simulate their application to larger populations.

In this paper, we present an example of how, through the collaboration between social psychology and AI, it is possible to develop personalized communication strategies which, precisely because such, have a better chance of being effective than the standard and one-sided communication that often characterizes the functioning of apps and other digital devices supporting people in their food choices.

We involved a sample of Italian participants in an experiment consisting of three steps. First, they filled out a questionnaire aimed at detecting the psychosocial antecedents of their intention to eat red/processed meat. Second, they read messages differing as to the framing of the hypothetical consequences of reducing (gain, non-loss) versus not reducing (non-gain, loss) RPMC (see also “Message Framing” section). Four different subgroups of the sample were randomly assigned to one of the four different framing conditions. Finally, all participants filled out a second questionnaire, which was aimed at detecting participants’ reaction to the messages they read, as well as any changes in their intention about RPMC. Data collected were then employed to learn both the structure and the parameters of a Graphical Causal Model (GCM) based on a Dynamic Bayesian Network (DBN), which was used for predicting the potential effects of message delivery from the observation of the psychosocial antecedents. Such probabilistic predictor is intended as the basis for developing automated interactions strategies using Deep Reinforcement Learning (DRL) techniques by which an artificial neural network can be trained toward a rapid estimate of the psychosocial antecedents and the automatic selection of the most effective message, according to the characteristics of the recipient.

In the remainder of this introduction, we will first examine the psychosocial models we referred to in the present research. Then, we will illustrate the reasons that led us to consider the application of Bayesian causal models as opportune and fruitful for the development of a personalized communication strategy.

### Message Framing

Past research has demonstrated that the persuasive effect of communication largely depends on how message recommendations are framed. Message framing refers to the fact that a given content can be presented in different, although objectively equivalent, versions (Davis, 1995; Chong and Druckman, 2007; Spence and Pidgeon, 2010). In the case of health recommendation messages, research on framing effects has widely focused on the differential effects of *valence framing*, that is, of messages framed either in terms of the *gain* deriving from following the proposed recommendations or the *loss* deriving from not following them (Gallagher and Updegraff, 2012; Rothman et al., 2020). The results of these studies have not been univocal, and this contradiction has been attributed, at least in part, to the fact that the distinction between gain and loss messages is not fine-grained enough (Dijkstra et al., 2011). In this vein, Cesario et al. (2013) have proposed a more fine-grained classification of valence framing, defined as the *outcome sensitivity* level of message framing. According to it, a message can motivate the receivers to adopt a healthy behavior through messages presenting the possible consequences of the behavior in four different ways: (a) *gain messages* state that *following* the recommendation will have a desirable consequence (e.g., “If you eat little red meat and cold cuts, you will improve the health of your stomach”); (b) *non-loss messages* state that *following* the recommendation will avoid having an undesirable consequence (e.g., “If you eat little red meat and cold cuts, you will avoid damaging the health of your stomach”); (c) *non-gain messages* state that *not following* the recommendation will imply missing the opportunity to have a desirable consequence (e.g., “If you eat much red meat and cold cuts, you will miss the chance to improve the health of your stomach”); (d) *loss messages* state that *not following* the recommendation will have an undesirable consequence (e.g., “If you eat much red meat and cold cuts, you will damage the health of your stomach”).

So far, some studies have considered the differential effects of gain, non-loss, non-gain, and loss messages on the promotion of physical activity (Catellani et al., 2021) and adherence to environmental policies (Bertolotti and Catellani, 2014). As to the effects of these four types of messages on the reduction of RPMC, Carfora et al. (2020b,2021) have showed that they differentially influence attitude and intention toward RPMC, and that this differential influence depends on some psychosocial characteristics of the receivers. These studies referred to the Theory of Planned Behavior (TPB; Ajzen, 1991) to identify some main antecedents of a change in the intention to eat red/processed meat after exposure to messages differing as to outcome sensitivity. Results showed that attitude, subjective norm, and perceived behavioral control measured before exposure to the messages did have an influence on whether and how far people changed their intention after message exposure. If TPB variables have been shown to contribute significantly to predicting intention change after message exposure, research on how to personalize message framing as a function of the recipients’ characteristics suggests that an in-depth analysis of what psychosocial dimensions may influence responses to different message frames is still a substantial research challenge (Rothman and Baldwin, 2019).

### Psychosocial Antecedents of Intention Change After Message Exposure

To move further in the comprehension of the dimensions that may underlie the effects of recommendation messages regarding RPMC, in the present study we considered not only TPB dimensions, but also several other psychological dimensions that previous research has shown to be related to RPMC and/or to the effectiveness of messages aimed at reducing the intention to eat red/processed meat. If research usually tests the influence of few dimensions at a time, thanks to the application of Bayesian causal models and clustering techniques, we were able to consider a considerable number of dimensions all together. We intended to predict which dimensions and which combinations of dimensions would lead to a change in the intention to eat red/processed meat, depending on the specific type of message received.

First, we considered the receiver’s *regulatory focus*. According to the Regulatory Focus Theory (RFT; Higgins, 1997), self-regulation with a *prevention focus* involves the avoidance of losses and the fulfillment of duties and obligations, while self-regulation with a *promotion focus* involves the pursuit of gains and the achievement of an ideal desirable state. Previous research has widely shown that the persuasiveness of a message increases when its framing matches the receivers’ regulatory focus (e.g., Yi and Baumgartner, 2009) and this is the case also for messages focused on healthy nutrition (Catellani et al., 2021).

Second, we considered differences in terms of *perceived severity* and *perceived susceptibility* related to health disease, factors that have proved to be important in people’s food choices. Specifically, people who believe that health diseases are threatening – In general, how likely do you think you will develop…that is when believing that they are susceptible (i.e., perceived susceptibility) to being affected by a harmful and severe disease (i.e., perceived severity) how severe you think is to develop gastro-intestinal diseases - intend less to act an unhealthy food choice (Urbanovich and Bevan, 2020).

Third, we introduced *food involvement* as another antecedent that could influence the processing and effectiveness of messages related to food choices. Previous research has shown that people who are more involved in food, that is, people who care about and are interested in food (Bell and Marshall, 2003), are more inclined toward making healthier food choices (Jezewska-Zychowicz et al., 2020). They could therefore be more interested in following the recommendation of a health message proposing reduction in RPMC.

Fourth, we know from previous research that people who strongly perceive that eating a specific food is risky (i.e., *perceived risk*) and that not eating it is beneficial (i.e., *perceived benefit*) tend to process nutritional messages more systematically and thereafter show lower intention to choose unhealthy food (e.g., Guo et al., 2020). Hence, we expected that receivers would respond differently to messages on RPMC reduction depending on their beliefs benefits and risks related to RPMC.

Fifth, we considered meat attachment (Graça et al., 2015) and moral disengagement (Graça et al., 2016) as dimensions that potentially would reduce the effectiveness of messages focused on RPMC reduction. As to *meat attachment*, we measured *hedonism*, that is, the perception that consuming meat is pleasant and a necessity in the diet (Verbeke, 2015; Graça et al., 2016). As to *moral disengagement*, we measured diffused responsibility, desensitization, and denial of negative consequences. People may decide to continue to eat meat because they believe that does not matter if they change their habits, since most people do not do the same. In this case, *diffused responsibility* occurs, which happens when people wait for someone else to make a moral decision. In the case of RPMC, the diffusion of responsibility makes people feel less pressure to reduce meat intake (Graça et al., 2016; Camilleri et al., 2020). *Desensitization* concerns the belief that the death and suffering of animals used for food purposes is a normal practice (Graça et al., 2016; Camilleri et al., 2020). It is related to the so-called meat dissociation, that is, mentally separating meat from its animal origins. Finally, the *denial of the negative consequences* regards the denial of the consequences that RPMC has on the environment, the public health, and animal welfare. Previous research has shown that denying the environmental impact of massive meat production leads people to a more negative evaluation of meat reduction and to increased intent to eat meat (Carfora et al., 2020a).

### Message Processing

Previous research testing the effects of differently framed messages focused on RPMC reduction has shown that message effectiveness largely depends on how messages are processed and evaluated (e.g., Carfora et al., 2019a,2020b). Consistent with previous research on persuasion (Petty and Cacioppo, 1986) these studies showed that the effect of the message on intention change was strongly affected by *systematic processing*, that is, the cognitive activity that takes place in response to a message. Two other dimensions that were shown to be related with message effectiveness were *message involvement*, that is, the degree to which the receiver perceives the message as interesting and involving (Karmarkar and Tormala, 2010), and *message-induced distress*, that is, the degree to which the receiver feels that the message stimulated uneasiness and fear (Brown and Smith, 2007). Starting from the above, in the present study we tested if systematic processing, message involvement, and message-induced distress would influence the effectiveness of gain, non-loss, non-gain, and loss messages promoting the reduction of people’s RPMC.

### Graphical Causal Model as a Probabilistic Predictor

To achieve an empirically effective model on how the psychosocial antecedents of RPMC interact with the message frame, we made use of AI techniques to construct a probabilistic predictor. Such achievement is intended to represent the first step in a process that generates automated interactions based on psycho-social models *via* AI. More precisely, the predictor should allow selecting the message framing that is most likely to be effective by relying on the observation, possibly even partial, of psychosocial antecedents alone.

To do so, we developed a method for eliciting a probabilistic graphical structure, that is, a (DBN; Dagum et al., 1995; Murphy, 2012) from experimental data. By design, the structure of the elicited DBN can be interpreted as a (GCM; Pearl, 2000) that allows estimating the *potential effects* of each message framing starting from the observation of the antecedents for a particular subject.

Overall, the purpose of our research is using the GCM as the basis for a wider system using DRL to generate automated policies for personalized interactions. The overview and the technical details of the DRL-based system can be found in Carfora et al. (2020b). In short, DRL is a technique for training a Deep Neural Network (DNN) which encodes an optimized *strategy* of action, as it could be for playing a game like chess or Go. Given the representation of an ongoing situation, a trained DNN can estimate what could be the long-term advantage of each possible action. In the context of this study, the aim of DRL was achieving a strategy for: (a) collecting information about the interactant using a modicum of questions; (b) selecting the intervention that is expected to be most effective, given the information acquired. The training process of DRL, however, involves reproducing a huge number of episodes, each like a match in a game, in which the machine can explore several alternatives and observe their effects.

In such scenario, a GCM-based probabilistic predictor acts as a simulator of the interactants, that is, by producing (stochastic) responses to machine actions. There are two further advantages in using a GCM as the basis for DRL. First, the probabilistic predictor can provide an effective and computationally manageable representation of the psychosocial models adopted. Second, the GCM can guarantee an explanation of the DRL policy, once the training process has been completed, in a way that the DNN alone could not provide (Linardatos et al., 2021).

### The Present Study

Starting from the above, in the present study we developed and tested a model to select messages focused on the reduction of RPMC and tailored on the characteristics of the recipient. In consideration of the type of analysis chosen, based on prediction and not on explanation, we did not formulate specific hypotheses, but some research questions (RQ) aimed at discovering whether the results of the predictive analysis would have validated the relevance of the psychosocial constructs described above in influencing the change of intention after exposure to messages. We therefore asked ourselves the following five main RQ:

RQ1 Does reading the recommendation messages used in the study (directly and/or indirectly) predict intention change regarding RPMC?

RQ2 Which psychosocial dimensions have a direct influence on intention change after exposure to recommendation messages?

RQ3 Which psychosocial dimensions have an indirect influence on intention change, and through the mediation of which message-related dimensions?

RQ4 Are there differences in the influence of the four message frames (gain, non-loss, non-gain, loss) on intention change depending on the characteristics of the receivers and, if so, what differences?

## Methods

### Participants and Procedure

The present study received ethical approval from the Catholic University of the Sacred Heart (Milan). Using the *A-priori* Sample Size Calculator for Structural Equation Models created by Soper (2021), we computed the minimum sample size required for a structural equation model study. Results recommended to involve at least 227 participants to test our full model (20 latent variables and 106 observed variables; expected effect size = 0.30; *p*-value = 0.05; statistical power level = 0.80) and 299 participants to test the moderation of the four levels of past adherence to the MeDiet (80 latent variables and 424 observed variables; expected effect size = 0.30; *p*-value = 0.05; statistical power level = 0.80). However, we opted to increase our sample size, following Jackson’s recommendation (Jackson’s, 2003) to have a sample-size to parameters ratio of 20:1 or at least 10:1. About 1,200 Italian citizens were invited to participate in a university study on the effects of public communication. They received an email with a link to an online questionnaire. An initial sample of 834 participants provided their informed consent and answered the questionnaire (Time 1-T1; see Measures in the “Time 1 Measures”). After that, participants who followed a specific diet (i.e., vegan, vegetarian, protein, slimming diets, *N* = 124), and those who weekly ate less than 3 portions of red and processed meat (*N* = 96) were eliminated from the sample. Then, participants were randomly assigned to one of four different experimental conditions (gain, non-loss, non-gain, and loss messages). One week after the completion of the first questionnaire, they were required to read eight messages focused on the health consequences of RPMC and framed according to the experimental condition they had been assigned to. Immediately after reading the messages, participants filled in a second questionnaire (Time 2-T2), assessing their evaluation of the messages, as well as their future intention to eat red/processed meat. The dependent variable *Delta intention* was then calculated as the difference between Intention measured at Time 2 and Intention measured at Time 1. Participants who did not fully or accurately complete both questionnaires were excluded (*N* = 70). Therefore, the final sample was composed by 545 participants (257 males and 288 females; mean age = 39.97, SD = 14.78; age ranging from 18 to 70 years), distributed in the four message conditions as follows: gain message condition *N* = 134; non-loss message condition *N* = 134; non-gain message condition *N* = 136; loss message condition *N* = 141.

### Time 1 Measures

In the first questionnaire, participants answered a series of questions measuring the relevant psychosocial dimensions focused on in the study on 7-point Likert scales.

*Attitude* toward reduced RPMC was measured using a semantic differential scale with eight items [e.g., “Eating little red/processed meat is… Bad (1)–Good (7)”; Carfora et al. (2017); Cronbach’s alpha, from now on α = 0.91].

*Subjective norm* was assessed with six items [e.g., “Most people who are important to me think that I should eat little red/processed meat… Strongly disagree (1)–Strongly agree (7)”; Carfora et al. (2017); α = 0.87].

*Perceived behavioral control* was assessed using nine items [“If I wanted, I’d be able to avoid eating eat red/processed meat when I am busy… Strongly disagree (1)–Strongly agree (7)”; adapted from Weller et al. (2014); α = 0.89].

*Baseline intention* to eat red and processed meat was assessed with three items on a seven-point Likert scale [e.g., “In the next month, how often do you intend to eat red/processed meat? … Never (1)–Every day (7)”; Carfora et al. (2017; α = 0.97)].

*Past behavior*, related to the quantity of RPMC eaten in the previous week, was assessed with two items: “Red meat includes all meat that turns dark when cooked and that is obtained from slaughter animals, such as veal, beef, pork, horse, kid, wild boar, deer, lamb. How many servings of red meat have you eaten in the past week?”; “Processed meat includes all meat that is subjected to processing processes, such as salting, seasoning, and smoking. It includes non-cured meats (for example, raw and cooked ham, bresaola, and speck) and sausages (for example, mortadella, sausage, and frankfurters). How many servings of processed meat have you eaten in the past week?” (Pearson correlation coefficient, from now on *r* = 0.48; *p* = 0.001).

*Prevention focus* was assessed using the nine prevention items of the 18-item General Regulatory Focus Measure [e.g., “I am anxious that I will fall short of my responsibilities and obligations… (1) Strongly disagree–(7) Strongly agree”; Lockwood et al. (2002; α = 0.75)].

*Promotion focus* was assessed using the nine promotion items of the 18-item General Regulatory Focus Measure [e.g., “I frequently imagine how I will achieve my hopes and aspirations… Strongly disagree (1)–Strongly agree (7)”; Lockwood et al. (2002; α = 0.83)].

*Perceived susceptibility* was measured with six items, introduced by “In general, how likely do you think you will develop… e.g., problems with the gastro-intestinal system (stomach, intestinal, pancreatic cancer, and digestive difficulties); problems with the cardiovascular system (heart attack and circulatory difficulties); metabolic problems (diabetes)… Extremely unlikely (1)–Extremely likely (7)”; adapted from Lea and Worsley (2002; α = 0.79).

*Perceived severity* was measured with six items [e.g., “In general, how severe you think is to develop gastro-intestinal diseases… Extremely severe (1)–Extremely severe (7)”; adapted from Lea and Worsley (2002; α = 0.88)].

*Food involvement* was measured with three items [e.g., “Food information is very relevant to me… Strongly disagree (1)–Strongly agree (7)”; adapted from Jung et al. (2016; α = 0.84)].

*Perceived risk* was measured with six items [e.g., “If you eat too much red/processed meat, how likely do you think you will develop gastro-intestinal diseases… Strongly disagree (1)–Strongly agree (7)”; adapted from Brown and Smith (2007; α = 0.91)].

*Perceived benefit* was measured with six items [e.g., “If you eat a little bit of red/processed meat, how likely do you think you will improve gastro-intestinal functioning… Strongly disagree (1)–Strongly agree (7)”; adapted from Brown and Smith (2007; α = 0.92)].

*Hedonism* was assessed using four items [e.g., “To eat meat is one of the good pleasures in life… Strongly disagree (1)–Strongly agree (7)”; adapted from Graça et al. (2015; α = 0.92)].

*Diffused responsibility* was assessed with three items [e.g., “Even if I change my habits, I don’t make a difference by myself… Strongly disagree (1)–Strongly agree (7)”; Graça et al. (2016; α = 0.87)].

*Desensitization* was measured using four items [e.g., “If I saw an animal being killed, I would have no problems eating it… Strongly disagree (1)–Strongly agree (7)”; Graça et al. (2015; α = 0.84)].

*Denial of negative consequences* was measured using five items [e.g., “To eat meat is disrespectful toward life and the environment… reverse item – Strongly disagree (1)–Strongly agree (7)”; adapted from Graça et al. (2016; α = 0.84)].

### Message Intervention

One week after completing the questionnaire at Time 1, all participants were contacted again and invited to read eight messages (approximately 14 words each) describing the health consequences of RPMC and varying according to the experimental condition. Participants in the *gain message condition* read messages on the positive health consequences associated with little RPMC (e.g., “If you eat little red meat and cold cuts, you will improve the health of your stomach”). Participants in the *non-loss message condition* read messages about how little RPMC prevents negative health outcomes (e.g., “If you eat little red meat and cold cuts, you will avoid damaging the health of your stomach”). Participants in the *non-gain message condition* read messages emphasizing how excessive RPMC is related to missing out on positive health outcomes (e.g., “If you eat much red meat and cold cuts, you will miss the chance to improve the health of your stomach”). Finally, participants in the *loss message condition* read messages about the negative health consequences of excessive RPMC (e.g., “If you eat much red meat and cold cuts, you will damage the health of your stomach”). The full list of messages is presented in the Appendix.

### Time 2 Measures

Immediately after reading the messages, participants were invited to fill out a second questionnaire to measure their reaction to the messages and possible change in their intention to eat red/processed meat.

*Systematic processing* was measured with five items, asking how deeply message information was processed [e.g., “I tried to think about the importance of the information for my daily life… Strongly disagree (1)–Strongly agree (7)”; adapted from Smerecnik et al. (2012; α = 0.87)].

*Message involvement* was measured with three items, asking participants to state their interest and involvement in the messages (e.g., “Messages were very interesting… Strongly disagree (1)–Strongly agree (7)”; adapted from Godinho et al. (2016; α = 0.91)].

*Message-induced distress* was measured with six items, asking whether participants had perceived uneasiness and distress upon reading the messages [e.g., “To what extent when reading these messages did you feel scared… Strongly disagree (1)–Strongly agree (7)?”; adapted from Brown and Smith (2007; α = 0.91)].

*Future intention* to eat red and processed meat was assessed with the same three items employed in the questionnaire completed at Time 1 (α = 0.78). A *Delta intention* variable was then calculated as the difference between Intention measured at Time 2 and Intention measured at Time 1.

The means and standard deviations of all variables at Time 1 and Time 2 are reported in Table 1.

**TABLE 1:** Means and standard deviations of the study measures in the four message conditions.

	Gain		Non-loss		Non-gain		Loss		Entire sample	
Measure	*M*	SD	*M*	SD	*M*	SD	*M*	SD	*M*	SD
**Time 1**										
Attitude	4.43	1.47	4.67	1.36	4.71	1.24	4.63	1.29	**4.61**	**1.34**
Subjective norm	3.50	1.45	3.60	1.38	3.64	1.36	3.48	1.42	**3.55**	**1.40**
Perceived behavioral control	4.09	1.20	4.18	1.19	4.48	1.16	4.18	1.17	**4.23**	**1.19**
Baseline intention	4.28	1.11	4.15	3.02	4.15	3.18	4.05	4.23	**4.16**	**3.10**
Past behavior	7.14	3.51	6.92	3.79	6.87	3.35	7.01	3.47	**6.99**	**3.52**
Prevention focus	4.50	1.04	4.63	0.99	4.73	0.92	4.70	0.92	**4.64**	**0.97**
Promotion focus	4.95	1.00	4.97	0.96	5.18	0.98	4.98	0.92	**5.02**	**0.97**
Perceived susceptibility	3.83	1.06	4.12	1.12	3.95	0.97	4.16	1.01	**4.02**	**1.05**
Perceived severity	4.16	0.63	4.14	0.64	4.17	0.69	4.18	0.57	**4.16**	**0.63**
Food involvement	5.27	1.15	5.31	1.18	5.12	1.21	5.39	1.00	**5.27**	**1.14**
Perceived risk	3.52	1.22	3.81	1.29	3.74	1.13	3.62	1.09	**3.67**	**1.19**
Perceived benefit	3.97	1.30	4.13	1.37	4.12	1.20	4.09	1.21	**4.08**	**1.27**
Hedonism	4.88	1.39	4.83	1.38	4.51	1.46	4.79	1.36	**4.75**	**1.40**
Diffused responsibility	3.61	1.16	3.77	1.27	3.74	1.20	3.61	1.18	**3.68**	**1.20**
Desensitization	3.70	0.50	3.68	0.60	3.64	0.64	3.61	0.57	**3.66**	**0.58**
Denial of neg. consequences	4.15	1.11	4.36	1.24	4.34	1.19	4.34	1.23	**4.30**	**1.19**
**Time 2**										
Systematic processing	4.27	1.34	4.61	1.08	4.24	1.30	4.41	1.25	**4.38**	**1.25**
Message involvement	4.09	1.50	4.36	1.33	3.84	1.63	4.11	1.38	**4.10**	**1.47**
Message-induced distress	1.55	0.61	1.69	0.71	1.65	0.72	1.66	0.69	**1.64**	**0.68**
Future intention	4.35	0.95	4.41	0.94	4.35	0.86	4.39	0.87	**4.38**	**0.90**

*Bold values represent the M = Mean; SD = Standard Deviation.*

### Rationale of the Analyses Performed

The main steps in the analytic method applied to the collected data for this study can be summarized as follows:

(1) Automatic elicitation of a DBN having the best combination of in-sample explanatory capability and out-sample predictive capability.

(2) Interpretation of the elicited DBN as a GCM for assessing the *potential effects* of the messages.

(3) Analysis of the GCM through the creation of a simulated population representing all possible Time 1 observations, using the combination of all *potential effects* as a soft clustering profile.

These steps will be further described in the following section, together with the psychological interpretation of the results.

## Results

### Elicitation of a Dynamic Bayesian Network Structure From Collected Data

The procedure adopted for the automatic elicitation of a DBN structure from collected data was essentially the same described in Catellani et al. (2021). Only a concise description of the procedure will therefore be given here. The procedure adopted was an automated search among all the possible DBN structures obeying to specific constraints allowing the causal interpretation that will be described in the next section. A weighted combination of two Area-Under-Curve (AUC) metrics, for in-sample and out-of-sample tests respectively, was used as evaluation function for selecting the preferred DBN structure.

A *Bayesian Network* ℬ=(*V*,*A*,*p*) (BN, Darwiche, 2009) is a directed acyclic graph where nodes *V* correspond to the random variables in the model, *p* is a joint probability distribution over the set of random variables, and each link *A*⊆*V*×*V* represents an oriented dependence relation among two random variables. Assuming that {*X*_1_,…,*X*_*n*_} is the set of all random variables in the model, the joint probability distribution *p* can be factorized as:


$$p\left(X_{1},\ldots ,X_{n}\right)=\prod_{i}p\left(X_{i}|\pi \left(X_{i}\right)\right)$$

where π(*X*_*i*_) is the set of *parents* of *X_i_*, i.e., the set of random variables whose representing nodes have an arc directed toward the node representing *X_i_*.

A (DBN, Dagum et al., 1995; Murphy, 2012) is a BN that also includes the representation of *time*, intended as a discrete sequence of instants. In a DBN:

• Each node is associated to a specific time instant.
• The same random variable may correspond to more than one node, at different times.
• All links must respect the orientation of time, either by connecting nodes at the same instant or by being oriented from a previous instant to a subsequent one.

**FIGURE 1:**
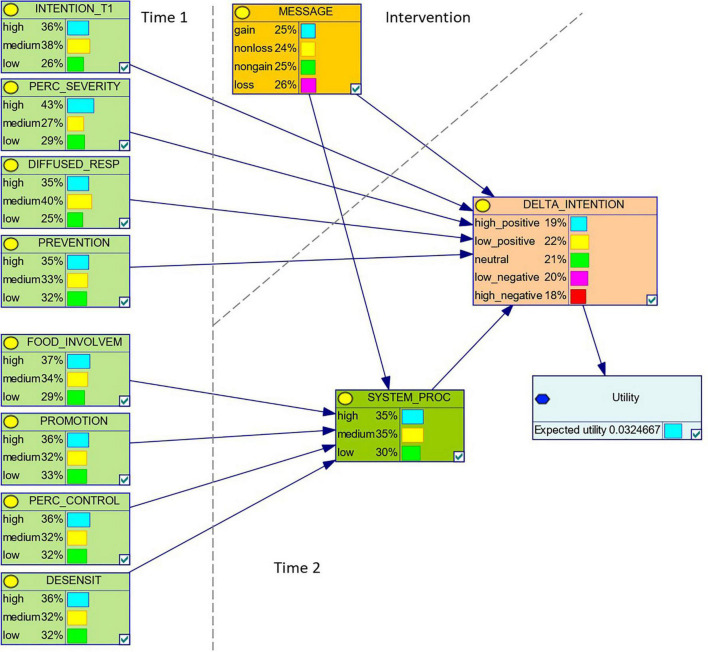
The Dynamic Bayesian Network selected by the automated structure elicitation procedure.

As it can be seen in Figure 1, in our study the DBN was assumed to span across a sequence of three instants: Time 1, message intervention, and Time 2.

For computational simplicity, the DBN was assumed to have categorical random variables only, in the scale {*low*, *medium*, *high*}, except for the target variable *Delta intention*, for which the scale {*high-negative*, *low-negative*, *neutral*, *low-positive*, *high-positive*} was adopted. Indexes were discretized using *quantiles*: 20% quantiles for *Delta intention* and 33% quantiles for all the other variables.

Our objective was to achieve a DBN that could predict the value of the target variable *Delta intention* relying only on Time 1 observations and message intervention, intended as the knowledge of the message framing selected. Calling *Z* the target variable for conciseness, the value predicted by the DBN will be:


$$z_{p\,r\,e\,d}:=\underset{\text{z}}{\text{argmax}}p\left(Z=z|Obs,Msg\right)$$

where *z* is one of the categorical values of *Z* and *p* is the probability computed by the DBN.

The baseline metrics adopted for evaluating the performances of candidate DBN structures is *Area Under Curve* (AUC; Fawcett, 2006), which measures the area under the curve traced by points:


(p(FP|γ),p(TP|γ))

where *FP* and *TP* are *False Positive* and *True Positive* value assignments, respectively, obtained when accepting a predicted value *z* whenever *p*(*Z*=*z*)≥γ, and varies in [0,1]. Such curve is also called *Received Operating Characteristic* (*ROC*). Given that the target variable in our case had five categorical values, in the present study the multiclass version of AUC (i.e., mAUC; Hand and Till, 2001) was used.

In-sample mAUC was estimated by learning probabilities from the dataset and then applying these same probabilities to each of the individuals in the dataset. Out-of-sample mAUC was estimated *via* the *leave-one-out*^1^ method (Raschka, 2018): one participant *d* is removed from the dataset *D*, then probabilities are learnt from (*D*−*d*) and predictive effectiveness is tested for participant *d* alone. The procedure was repeated for all participants in *D* and the resulting success ratio was computed. Given that, in general, in-sample performances are better than out-of-sample ones, the metrics adopted in this study was defined as:


$$m^{\left(\text{i}\right)}=\frac{{\text{mAUC}}_{\text{in}}^{\left(\text{i}\right)}}{\max_{\text{j}}\left({\text{mAUC}}_{\text{in}}^{\left(\text{j}\right)}\right)}+\frac{{\text{mAUC}}_{\text{out}}^{\left(\text{i}\right)}}{\max_{\text{j}}\left({\text{mAUC}}_{\text{out}}^{\left(\text{j}\right)}\right)}$$

where *m*^(*i*)^ is the performance measure for candidate *i* and the two denominators are the maximal values attained in all candidate DBN structures considered.

Given that computing the mAUC is expensive (especially in the leave-one-out case), the automated structure elicitation procedure was based on a preliminary screening to select the most promising candidate structures. In such screening, all random variables in the model were considered in sequence, starting from the target node and proceeding backward along the guidelines imposed by DBN modeling. In each step, the best parent sets for each random variable were selected according to their *information gain*. Let *X* be a random variable and {*Y*_1_,…,*Y*_*n*_} a set of possible parents, the information gain is defined as:


$$IG\left(X;Y_{1},\ldots ,Y_{n}\right):=H\left(X|Y_{1},\ldots ,Y_{n}\right)-H\left(X\right)$$

where *H* is the *entropy*:


$$H\,\left(X\right):=-\sum_{X}p\,\left(X\right)\,\log p\,\left(X\right)$$

and the *conditional entropy* is defined as:


$$H\left(X|Y_{1},\ldots ,Y_{n}\right):=-\sum_{X,Y_{1},\ldots ,Y_{n}}p\left(X,Y_{1},\ldots ,Y_{n}\right)\log\frac{p\,\left(X,Y_{1},\ldots ,Y_{n}\right)}{p\,\left(Y_{1},\ldots ,Y_{n}\right)}$$

In all the above equations^2^, *p* can be construed as the empirical probability distribution, estimated as frequency ratios in the dataset.

Once the preliminary screening was completed, for each selected candidate structure, we computed the *m*^(*i*)^ metric and the DBN scoring the highest value was selected. Further details about the procedure can be found in Catellani et al. (2021).

The DBN thus obtained is shown in Figure 1. The DBN was then trained using collected data, to compute the joint probability distribution. The complete definition of the DBN, together with the dataset used for structure elicitation and training, can be found at https://bitbucket.org/unipv_cvmlab/framing-and-tailoring-prefactual-messages-to-reduce-rpmc/.

### Interpreting the Elicited Dynamic Bayesian Network

The DBN structure that emerged from the automated analysis (Figure 1) allowed us answering our first three RQ about whether reading the recommendation messages would (directly and/or indirectly) predict intention change regarding RPMC (RQ1), as well as which psychosocial dimensions would be more (directly or indirectly) predictive of intention change (RQ2 and RQ3). Answering our RQ1, the DBN showed that message framing predicted intention change both directly and through the mediation of systematic processing. As to the answer to our RQ2, in addition to Intention at Time 1, other three psychosocial dimensions directly predicted intention change, and they were prevention focus, perceived severity, and diffused responsibility. As to our RQ3, promotion focus influenced intention change through the mediation of systematic processing, and the same happened for food involvement, perceived behavioral control, and desensitization.

These results were consistent with our expectation according to which exposure to prefactual messages would have increased the likelihood of participants reducing their intention to consume red/processed meat. At the same time, the DBN automatically selected as maximal offered several indications as regards which psychosocial dimensions are more likely to influence intention change regarding RPMC and whether such influence is more likely to be direct or mediated by the systematic processing of prefactual messages on RPMC reduction. The related theoretical implications are discussed in the section “Discussion.”

### Graphical Causal Model

From a structural viewpoint a GCM; Glymour et al., 2019) is identical to a Bayesian Network. The main difference is that arcs among nodes in the former have an intended *causal* meaning whereas in the latter they denote just dependencies. In other words, oriented arcs in a GCM imply that a change in the originating variable causes an effect in the destination one whereas the same does not occur in the opposite direction.

**FIGURE 2:**
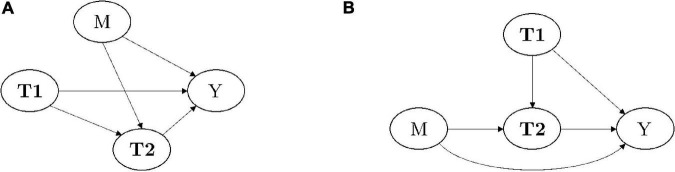
General structure of the DBNs in this study [**(A)** – see also Figure 1] and its interpretation as GCMs **(B)**. In the diagrams, boldface letters denote a set of random variables.

By design, any model in the class of DBNs of reference for this study can be construed as a GCM. Figure 2A shows the general structure of these DBNs: nodes **T1** stands for Time 1 dimensions being estimated and node M for the type of messages being sent in the experiment. Given that the choice of message framing is randomized, there is no dependence among the two. The main intervention is M, which causes the effects that can be estimated as Time 2 dimensions (node **T2**). By assumption, some of Time 1 dimensions may have influence on Time 2 dimensions. Finally, Time 1 dimensions, message framing, and Time 2 dimensions all have a direct influence on the measurable outcome *Y*, which in this study is related to the target variable *Delta intention*.

The graph shown in Figure 2B is a rearrangement of the graph in (A) and emphasizes the two main causal paths of interest in this study: one *direct* path M → Y and another *indirect* path M → **T2** → Y. The aim is identifying the causal effect of messages M onto the estimated outcome Y. From Figure 2B, it is evident that Node **T1** represents the *adjustment set* (Pearl, 2010) that must be made for estimating the *potential effects* of messages along the causal paths of interest. As a matter of fact, Node **T1** blocks all the unwanted back-door paths between M and Y. Here, potential effects are intended as those that could be induced in a subject, should it be possible to observe the outcome of each of them, as if they were sent to them in separate, *counterfactual* episodes.

Our declared intent was achieving a *predictor* for message effects that would allow selecting the message framing that would be more likely to produce a positive effect. It follows that, at the time of the choice of message type M, only **T1** variables might be observed. Observations may be complete or not, in the sense that some of the Time 1 dimensions might not have been estimated yet, for brevity of interaction. Formally:


$$\text{T}\,1={\text{T1}}_{o}\cup {\text{T1}}_{n}$$

where **T1**_*o*_ is the set of *observed* **T1** variables while **T1**_*n*_ is the set of *unobserved* variables. Therefore, the *average potential effects* (APE) we are interested in can be estimated as:


$$\text{E}\left[Y\left(m\right)|{\text{T1}}_{o}\right]=\sum_{y}yp\left(y|\mathrm{do}\left(m\right),{\text{T1}}_{o}\right)$$

In other words, under the principles of GCMs, the APE of a message framing *m* is the expected value of the outcome *Y* given potential interventions do(*m*) and the observations T1_*o*_. In the case of GCM structures described in Figure 2B, due to the so-called Back-Door Adjustment Theorem (Pearl, 2000), the APE becomes:


$$\text{E}\left[Y\left(m\right)|{\text{T1}}_{o}\right]=\sum_{y}y\sum_{{\mathrm{T1}}_{n}}p\left(y|m,{\text{T1}}_{o},{\text{T1}}_{n}\right)p\left({\text{T1}}_{n}\right)$$

For the sake of simplicity, in evaluating the results presented below we assumed that all Time 1 dimensions were observed, so that **T**1=**T1**_*o*_. In this case, APE can be estimated as:


$$\text{E}\left[Y\left(m\right)|\text{T}1\right]=\sum_{y}yp\left(y|m,\text{T}1\right)$$

In our study, the outcome *Y* was measured by a *utility function* intended to estimate to what extent the probability distribution over the target random variable was skewed in the desired direction. Formally:


$$Y\left(m\right):=\sum_{z}w\left(z\right)p\left(Z=z|m,\text{T}1\right)$$

where *Z* is the target random variable *Delta intention* and where the weighting function *w* assigns the values 2, 1, 0, -1, -2 to the categorical values *high-positive*, *low-positive*, *neutral*, *low-negative*, *high-negative*, respectively.

### Analyzing the Graphical Causal Model Through Soft Clustering

Interpreting a graphical model, be it a GCM or a DBN, with all probability parameters learnt from data, is difficult. A completely defined GCM describes a joint probability distribution over the entire set of random variables in which the influences on each variable by other ones are represented as numerical definitions of conditional probabilities. Nonetheless, such distributed representations must be considered as one, when analyzing information conveyed by GCM. To make the potential effects described more readable, we created a simulated population including one individual per each possible configuration of values for the categorical variables in **T1**. In the model presented in Figure 1 there are 8 **T1** variables that can assume 3 categorical values each. Therefore, the simulated population created contained 3^8^=6561 individuals. For each individual, we computed the APE of each message framing *m*. In this way, we could associate each individual to a four-dimensional numerical vector:


$$\left(e_{g\,a\,i\,n},e_{n\,o\,n\,l\,o\,s\,s},e_{n\,o\,n\,g\,a\,i\,n},e_{l\,o\,s\,s}\right)$$

where each value *e_m_* represents the estimated APE of message framing *m*. In this way, each 4D vector can be construed as the soft clustering profile of the corresponding individual. Clustering diagrams can be traced by assuming the Euclidean distance among 4D vectors as a dissimilarity measure among individuals.

**FIGURE 3:**
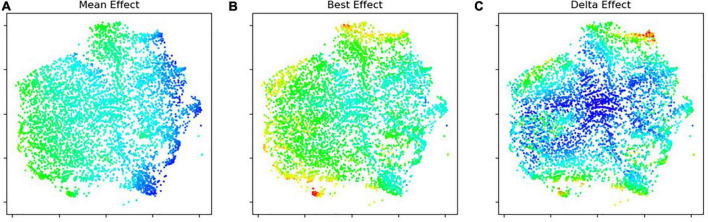
Clustering diagrams made with T-SNE using potential effects on a simulated population. Coldest colors represent lowest values: mean message effect **(A)**; best message effect **(B)**; delta between best message effect and mean message effect **(C)**.

Figure 3 shows the result of applying the t-SNE algorithm (van der Maaten and Hinton, 2008) to plot all the 4D vectors from the simulated population into a 2D diagram, while respecting as much as possible the relative distances among them. In the three diagrams (A–C) in Figure 3 the spatial positioning is the same. Colors in each diagram represent numerical values form each 4D vector, namely: (A) the *mean effect*, intended as the average of the four values; (B) the *best effect*, intended as the pivot in each vector; (C) *delta effect*, intended as the difference between the pivot and the mean message effect. In these versions, the coldest color (deep blue) represents minimal values while the warmest color (bright red) represents maximal ones. Color scales are identical in (A,B), with deep blue representing negative values, while in diagram (C) deep blue represents zero. As it can be seen, individuals in which the effects are more pronounced, either positively or negatively, tend to occupy spatial positions on the border of the main cluster.

**FIGURE 4:**
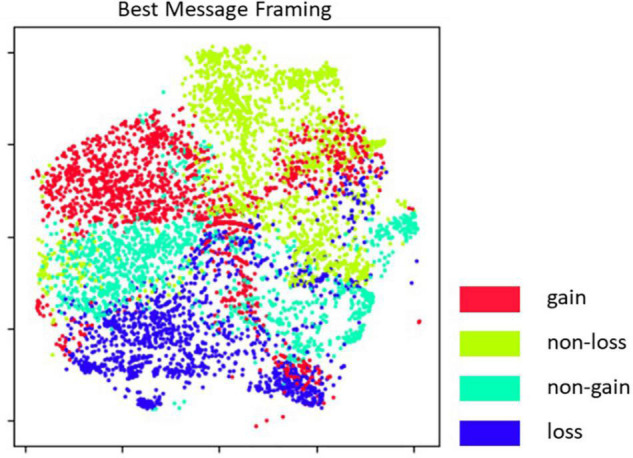
Clusters of individuals by the most effective message framing. Spatial positioning of the simulated population is the same as in the previous figure.

Figure 4 shows the sub-clustering of individuals whereby colors correspond to the most effective message framing, as denoted by the pivot in each 4D vector. The spatial positioning is the same as in Figure 3. As it could be expected, individuals scoring higher best and delta effects combined also tend to be better characterized in terms of which framing might be more effective.

### Interpreting Clustering Prototypes

Using the soft clustering profile, we also extracted a few relevant prototypes in the joint probability distribution. By selecting the 2% quantile of the highest best message effects, we identified the prototypical receptors for each the four message framings *gain*, *non-loss*, *non-gain*, and *loss* respectively. The prototype of *indifferent* receptors, namely those individuals that may be receptive to messages but are not particularly affected by message framing was selected by considering the 2% quantile of the lowest delta effects. *Oppositive* prototypical receptors, namely those who are expected to react negatively to messages, regardless of framing, were identified by selecting the 2% quantile of the lowest mean effects.

We employed the results of the above soft clustering to answer our RQ4 regarding which combinations of psychological antecedents would lead participants to prefer a given message frame rather than another, changing their intention to eat red/processed meat accordingly. We first distinguished participants according to their high, medium, or low intention to eat red/processed meat at Time 1, that is, before being exposed to the messages. We then considered two other relevant and direct antecedents of Delta intention after message exposure, namely, prevention focus and the perceived severity attributed to the probability of developing diseases. The results of the analysis showed that different combinations of these three dimensions led to identify seven prototypes, who are presented in Table 2 and described below.

**TABLE 2:** Best message framing as a function of prototypes emerged from clustering analysis.

Prototype	Intention to eat red/processed meat	Prevention focus	Perceived severity	Best framing
Strong meat eater 1	High	High	High	Loss
Strong meat eater 2	High	High	Low	Indifferent
Medium meat-eater 1	Medium	Medium	High	Gain
Medium meat-eater 2	Medium	Low	High	Non-gain
Medium meat-eater 3	Medium	Low	Low	Non-loss
Low meat-eater 1	Low	High	High	Non-loss
Low meat-eater 2	Low	Low	Low	Oppositive

*Indifferent: participants tend to be equally persuaded by all message frames; oppositive: participants are not persuaded by any messages.*

In the case of strong meat eaters, namely, participants with a high intention to eat red/processed meat, two main prototypes emerged:

- **Strong meat-eater 1.** They want to eat a lot of meat, but at the same time they are prevention-oriented and consider health diseases as very severe. They are more persuaded by **loss messages**. Therefore, they are convinced by messages stressing the negative health consequences deriving from eating too much meat.

- **Strong meat-eater 2.** They want to eat a lot of meat, are prevention-oriented but not especially worried by diseases. These participants tend to be equally persuaded by all messages. They may therefore be considered **indifferent** as regards frame. This result suggests that prevention-oriented participants who are not especially scared by diseases can follow a health recommendation independent from how it is formulated.

In the case of medium meat-eaters, namely, participants with a medium intention to eat red/processed meat, we obtained three prototypes:

- **Medium meat-eater 1**. They have an intermediate level of prevention focus but consider diseases as very severe. They are especially persuaded by **gain messages**. This finding means that informing on gaining positive health outcomes is effective for people who do not considerably intend to eat meat, perceive health diseases as severe but are not strongly prevention oriented.

- **Medium meat-eater 2**. They have a low level of prevention focus, but they consider diseases as very dangerous. They are persuaded by **non-gain messages**. This finding suggests that providing information on missing out on positive health outcomes is effective for low prevention-oriented people who are more sensitive to losing positive health consequences, as they fear getting sick.

- **Medium meat-eater 3**. They have a low level of prevention focus and do not consider diseases as dangerous. They are more persuaded by **non-loss messages**. This finding means that informing on avoiding negative health outcomes is effective for people who do not considerably intend to eat meat and prefer a growth health goal that allows them to avoid negative consequences.

Finally, in the case of low meat-eaters, namely, participants with a low intention to eat red/processed meat before the intervention, we identified two prototypes:

- **Low meat-eater 1**. They are strongly prevention-oriented and consider health diseases as very severe. They are persuaded by **non-loss messages**. Therefore, individuals who already had a low intention to eat meat, were strongly prevention-oriented and scared by diseases prefer to receive information about what negative consequences they will avoid if they pursue their healthy intention.

- **Low meat-eater 2.** They are not prevention-oriented and consider diseases as not so severe. These individuals are **oppositive**, that is, they are not persuaded by any messages. This result suggests that individuals who already have a low intention to eat meat, have a low prevention focus and are not scared by diseases are unlikely to be convinced to further reduce their RPMC by health recommendations, independent on how they are framed.

## Discussion

Thanks to the integration of theoretical models developed in the field of social psychology and the possibilities of probabilistic calculation allowed by AI, in this study we have developed a procedure to personalize messages aimed at favoring the reduction of RPMC, and thereby make them more effective. We identified psychological factors that play an important role in bringing about a different RPMC change after being exposed to differently framed messages. What we found constitutes an advancement of our knowledge both in terms of matching between message framing and message target in the field of dietary recommendations and in how AI can contribute to the development of increasingly effective communication strategies, extensible through the network to a very large number of people.

The analysis of the GCM obtained through the simulated population supported our expectation that message exposure would predict intention change both directly and through the mediation of systematic processing. Consistent with previous research (Carfora et al., 2021), when the messages induced further thoughts and reflections, they were also more likely to predict intention change. The analysis of the GCM also made it possible to identify which of the different psychological dimensions that weigh on the choice to consume red/processed meat are more likely to influence people exposed to messages with different frames in terms of outcome sensitivity (Carfora et al., 2020b). While some dimensions are more likely to have a direct influence on intention change, the effect of some other dimensions is more likely mediated by the fact of processing the message in a systematic way.

In addition to Intention at Time 1, there were three other *direct* predictors of intention change. One of them was prevention focus. Interestingly, prevention focus had a direct link with intention change, while this was not the case for promotion focus, which had only a link mediated by systematic processing. This evidence suggests that prevention-focused people (i.e., those who are oriented to avoid losses) are influenced by the health-related messages on red/processed meat reduction regardless of how deeply they processed the messages (for a similar result, see Lalot et al., 2019). Evidently, people who have a prevention goal are likely to immediately capture the meaning of messages that explain the health consequences of eating or not eating meat, given that such information is consistent with their goal of avoiding health diseases. On the one hand, these results are consistent with previous research showing that the effect of differently framed messages varies according to the recipient’s regulatory focus (Latimer et al., 2008; Pfeffer, 2013). On the other hand, they are also consistent with previous research suggesting that prevention-oriented people are often more strongly influenced by message framing than promotion-oriented people (Bertolotti and Catellani, 2014; Bertolotti et al., 2020).

The severity participants attributed to the possibility of developing diseases was another direct predictor of Delta intention after message exposure. This result is consistent with previous studies in the domain of health and eating behavior (e.g., Napper et al., 2014), as well as with scientific evidence showing that the effectiveness of message framing depends on the nature of the health behavior focused on and the risk ratio of its complications (Zareharofteh and Karimi, 2021). A moral disengagement variable such as diffused responsibility also had a direct link with delta intention. Evidently, perceiving that the reduction of meat consumption is useless because the majority does not do that has a direct influence not only on the intention to reduce RPMC (Graça et al., 2016), but also on intention change after exposure to messages focused on such reduction.

If we now turn to the dimensions which influenced intention change through the mediation of systematic processing, we find four psychological dimensions. Apart from promotion focus, already commented above, the other three dimensions were food involvement, perceived behavioral control, and desensitization. Not surprisingly, being interested and involved in food-related information led to systematic processing of the messages, consistent with the results of previous persuasion research (Jezewska-Zychowicz et al., 2020). The more people were interested in receiving information regarding healthy eating, the more they carefully elaborated the messages that provided food and health-related information, and this led to intention change.

Perceived behavioral control also had an influence on intention change, through the mediation of systematic processing. This result is consistent with previous research showing that persuasive messages on nutrition are in general more convincing for recipients with high self-efficacy (a proxy of perceived behavioral control; Riet et al., 2008; Bertolotti et al., 2020). People who feel that they have the necessary skills to perform the message recommendations are motivated to accept it and change their behavior accordingly. Conversely, people who feel they are not able to deal with the requests tend to activate defense mechanisms that lead them to simply ignore or reject the threatening message. In the present study, however, the predictive DBN suggested that the role played by perceived behavioral control in influencing message effectiveness may depend on the participant engaging or vice versa not engaging in a systematic processing of the messages received. A superficial elaboration of the messages, possibly due to low interest in the issue dealt with in the messages, can therefore reduce (or even reverse) the link between perceived behavioral control and intention change after message exposure.

Finally, a moral disengagement dimension such as receivers’ desensitization to the death and suffering of animals used for food purposes influenced intention change through systematic message processing. We know from previous research that people often dissociate meat from its animal origins to deal with the cognitive dissonance resulting from liking meat but disliking causing pain to animals. The importance of considering desensitization as a key predictor of people’s changes in the intention to eat meat is supported by past results showing that individuals, when consuming meat, tend to reduce their attributions of mind and sentience to animals, limiting the moral relevance of eating meat (Mathur et al., 2020).

To sum up, the probabilistic causal structure that emerged was in keeping with the relevance of the psychosocial models we referred to as a starting point of the analysis. At the same time, it showed the predictive weight of the considered dimensions differed, with only the most influential dimensions appearing in the final predictor. More specifically, it confirmed the opportunity to consider, in addition to TPB dimensions considered in the previous studies (Di Massimo et al., 2019; Carfora et al., 2021), other dimensions that contributed to increase the predictive power of the model as regards intention change after message exposure. Regulatory focus and individual differences in the perceived severity of the diseases that can be contracted were important predictors of the intention to reduce RPMC. On the contrary, diffused responsibility with respect to the consequences for the environment of eating too much meat and desensitization with respect to the pain of animals [i.e., two of the dimensions included in Graça et al. (2015) model of moral disengagement] proved to be particularly relevant in “rowing against” the possibility to change intention toward a reduced RPMC.

The most precise and useful indications for understanding the characteristics of the probabilistic predictor emerged from the soft clustering analysis. Such an analysis paves the way to tailoring the messages to be sent to people regarding the reduction of RPMC. The outcome of the analysis suggested the opportunity to distinguish between those who have a low, medium, or high RPMC. Within each category, the clusters that emerged as the most discriminating were characterized by different combinations in terms of high-medium-low prevention focus and perceived disease severity. In this way it was possible to identify the prototypes of people who are more likely to change after being exposed to gain, non-loss, non-gain, and loss messages, respectively.

The two profiles that emerged both in the case of strong meat-eaters and in the case of low meat-eaters were particularly interesting. As for strong meat-eaters, if a high focus on prevention is accompanied by a high concern for the severity of diseases, a significant effect of loss messages is more likely, confirming the fact that for these people the register of negativity appears crucial. On the other hand, when the concern for diseases is low, even in the presence of a high orientation toward prevention, a general sensitivity to messages is observed regardless of their framing. The difference between this and the other profile could be traced back to a higher degree of pathogen avoidance (Neuberg et al., 2011) and regulation of negative emotions in the case of the latter profile. The sensitivities to messages appear very different in the case of the two low meat-eaters profiles. Prevention-oriented low meat-eaters worried about disease are particularly stimulated to change after reading non-loss messages. These messages appear consistent with a path of avoiding the consumption of meat that these people have probably already undertaken for some time and that puts them in a less “dramatic” condition compared to strong meat-eaters. The second profile that emerged among low meat-eaters is the one of people who, for reasons not linked to a strong concern for diseases, already consume little meat. These people are evidently bothered by messages that insist on a goal that is probably already achieved and not so relevant to them.

In short, our analysis confirmed the opportunity of profiling people in depth to identify the messages most in tune with them. It also allowed to identify the prototype of people who are more likely to be convinced by the messages regardless of their framing was also identified, as well as of people who show themselves resistant to all messages, again regardless of their framing. More generally, our analysis further supported the usefulness of using a prefactual (i.e., “if… then”) style in messages aimed at supporting behavior change (Bertolotti et al., 2016). As compared to other more direct messages, prefactual messages decrease the reactance that more direct messages can arouse (Bertolotti et al., 2020). They also allow people to anticipate the outcomes of possible future actions, thus favoring the initiation of a change process (Petrocelli et al., 2012).

What emerged from our study offers some suggestions for the development of effective communication campaigns on RPMC reduction. First, the opportunity to develop and use prefactual messages is further supported by our data. At first glance, prefactual messages may seem less effective than more direct messages, as they are less strong from the illocutionary point of view. However, these messages, precisely because of their being indirect and therefore less overtly persuasive, often result very effective (Bertolotti et al., 2016). Second, a careful modulation of message framing according to the characteristics of the recipient is clearly opportune, not only to increase the effectiveness of a campaign, but also to avoid oppositive reactions, which may even be counterproductive with respect to the desired purpose of the campaign (Wolstenholme et al., 2021). As we have seen, some characteristics of the recipients, such as how much meat they eat, but also a prevention focus or their level of concern for diseases have special relevance in this case. In real life it is not always possible to have this information about recipients in advance. However, this information might at least partially be inferred from the context in which the campaign takes place, from socio-demographic characteristics of the target people, or even their online behavior. The network offers many opportunities from this point of view and the traces we leave online may be used not only (as it is often the case) for the sale of products, but also for the tailoring of messages that can support people in their search for health and well-being (all this obviously in full transparency and respect of privacy).

Our research has some limitations, related to some characteristics of the sample used and the procedure followed in data collection. The sample was not fully representative of the population and replicating the survey with another sample would allow us to subject the model that emerged from our analysis to further validation. As for the research procedure, people read the eight messages all together and replied about their intention to consume red/processed meat immediately thereafter. Further studies that involve sending multiple messages over a period of two or more weeks could be useful to further corroborate the results of the predictive model (Carfora et al., 2019b). Moreover, the health consequences described in the messages we used in this study were rather generic. This was functional for us to use short and simple prefactual sentences, but at the same time this choice might have reduced the perceived danger of what was said in the messages. Further studies might usefully be preceded by a pre-test assessing the comprehensibility or perceived danger of the messages employed. Similarly, a follow-up could serve as a check on the stability of any intention change that emerged. Finally, further studies could introduce further measures of change in nutritional habits after message exposure, for example by asking people to journal or photograph the foods they ate for a certain amount of time.

Beyond the afore mentioned limits, what has been achieved in this research opens the way to further steps in the implementation of an automated predictor. In keeping with our preliminary experiments, an artificial neural network can be trained using DRL techniques to guide interactions toward a rapid estimate of the psychosocial antecedents of intention change, as well as the automatic selection of the most effective messages, according to the characteristics of the recipient. This leads to the possibility to adapt interaction strategies according to people’s reactions and through a continuous learning process.

The goal of this path of integration between social psychology and AI is to develop communication on food choices that is on the one hand more and more personalized and in tune with the needs and resources of each individual, on the other hand aimed at an increasing number of people. The entire process can be based on consolidated theoretical models, as well as on monitoring and supervising machine learning, thus maintaining the high levels of transparency and explainability required of an AI truly at the service of the human.

## Data Availability Statement

The datasets presented in this study can be found in online repositories. The names of the repository/repositories and accession number(s) can be found below: https://bitbucket.org/unipv_cvmlab/framing-and-tailoring-prefactual-messages-to-reduce-rpmc/.

## Ethics Statement

The studies involving human participants were reviewed and approved by the Ethics Committee of the Catholic University of the Sacred Heart – Department of Psychology. The patients/participants provided their written informed consent to participate in this study.

## Author Contributions

PC proposed the research questions, supervised the conception, research design, interpretation of the data, and also thoroughly revised this manuscript as regards contents and style. VC proposed the research questions, planned the research design, and took responsibility for the data collection. MP proposed the research questions, analyzed the data, authored all software procedures, and supervised the interpretation of the results. All authors contributed to the article and approved the submitted version.

## Conflict of Interest

The authors declare that the research was conducted in the absence of any commercial or financial relationships that could be construed as a potential conflict of interest.

## Publisher’s Note

All claims expressed in this article are solely those of the authors and do not necessarily represent those of their affiliated organizations, or those of the publisher, the editors and the reviewers. Any product that may be evaluated in this article, or claim that may be made by its manufacturer, is not guaranteed or endorsed by the publisher.

## Footnotes

1 In the context of this study, also *leave-one-subject-out* would be an appropriate method designation.

2 In summations like the ones above, we adopt the shorthand notation of using random variables (e.g., *X*) as representatives of the possible values that these could assume.
